# A MultiTEP platform-based epitope vaccine targeting the phosphatase activating domain (PAD) of tau: therapeutic efficacy in PS19 mice

**DOI:** 10.1038/s41598-019-51809-2

**Published:** 2019-10-29

**Authors:** Armine Hovakimyan, Tatevik Antonyan, Sepideh Kiani Shabestari, Olga Svystun, Gor Chailyan, Morgan A. Coburn, William Carlen-Jones, Irina Petrushina, Jean Paul Chadarevian, Karen Zagorski, Nikolai Petrovsky, David H. Cribbs, Michael G. Agadjanyan, Anahit Ghochikyan, Hayk Davtyan

**Affiliations:** 1 Department of Molecular Immunology, Institute for Molecular Medicine, Huntington Beach, CA USA; 2 Sue and Bill Gross Stem Cell Research Center, University of California, Irvine, CA USA; 3 Department of Neurobiology and Behavior, University of California, Irvine, CA USA; 4 Institute for Memory Impairments and Neurological Disorders, University of California, Irvine, CA USA; 5 Vaxine Pty Ltd, Flinders Medical Center, Bedford Park, Adelaide 5042 Australia; 6 Department of Diabetes and Endocrinology, Faculty of Medicine, Flinders University, Adelaide, 5042 Australia; 7 Present Address: Department of Pharmaceutical Sciences, College of Pharmacy, University of Nebraska Medical Center, Omaha, Nebraska USA

**Keywords:** Immunotherapy, Neuroimmunology

## Abstract

Pathological tau correlates well with cognitive impairments in Alzheimer’s disease (AD) patients and therefore represents a promising target for immunotherapy. Targeting an appropriate B cell epitope in pathological tau could in theory produce an effective reduction of pathology without disrupting the function of normal native tau. Recent data demonstrate that the N-terminal region of tau (aa 2-18), termed the “phosphatase activation domain (PAD)”, is hidden within native Tau in a ‘paperclip’-like conformation. Conversely, PAD is exposed in pathological tau and plays an essential role in the inhibition of fast axonal transport and tau polymerization. Thus, we hypothesized that anti-tau2-18 antibodies may safely and specifically reduce pathological tau and prevent further aggregation, which in turn would neutralize tau toxicity. Therefore, we evaluated the immunogenicity and therapeutic efficacy of our MultiTEP platform-based vaccine targeting tau2-18 formulated with Advax^CpG^ adjuvant (AV-1980R/A) in PS19 tau transgenic mice. The AV-1980R/A induced extremely high antibody responses and the resulting sera recognized neurofibrillary tangles and plaque-associated dystrophic neurites in AD brain sections. In addition, under non-denaturing conditions AV-1980R/A sera preferentially recognized AD-associated tau. Importantly, vaccination also prevented age-related motor and cognitive deficits in PS19 mice and significantly reduced insoluble total and phosphorylated tau species. Taken together, these findings suggest that predominantly targeting misfolded tau with AV-1980R/A could represent an effective strategy for AD immunotherapy.

## Introduction

Alzheimer disease (AD) is the leading cause of age-related dementia, affecting 5.7 million people in the United States. Major challenges in AD include the lack of effective treatments, reliable biomarkers, or preventive strategies^1,2^. Unfortunately, several promising drug candidates have failed in clinical trials^3,4^ including the recently announced halt of a phase 3 trial of the beta-amyloid-targeting antibody aducanumab^5^. Hence, there is an urgent need to develop new prophylactic and therapeutic approaches to treat AD.

To develop potential disease-modifying drugs for AD, scientists have focused primarily on amyloid-β pathology^6,7^. Several active vaccines and monoclonal antibodies targeting Aβ have been tested in patients with mild-to-moderate AD with mediocre outcomes. However, some encouraging data from early phase 1 and 2 trials suggest that Aβ immunotherapy initiated at early stages (stages 2–3) of disease or even in healthy people at risk of AD, may provide some preventative benefits^8^. If that is the case, immunogenic and safe Aβ active vaccines may offer a promising approach for prophylactic use. On the other hand, new approaches and targets are being investigated and tau pathology is becoming one of the most promising new therapeutic targets^9,10^. Furthermore, although levels of Aβ oligomers correlate with disease severity^11–14^, tau pathology more strongly correlates with the progression of AD^9,15^. Based on these data, we hypothesize that tau targeting immunotherapy should be applied either simultaneously with anti-Aβ therapy as a prophylactic measure or sequentially in the later stages of AD.

Recently, several active and passive anti-tau immunotherapy strategies have been successfully tested in animal models^16,17^. Anti-tau active immunotherapy strategies are based on induction of high titers of antibodies capable of clearing pathological tau species that in turn would be expected to improve neuronal function and survival. Therefore, identifying an appropriate B cell epitope in tau is a key factor for a successful immunotherapeutic approach. Hyperphosphorylation has been considered as a strong candidate for inducing tau aggregation and neurofibrillary pathology, therefore, several vaccines targeting phospho-epitopes of tau have been developed and tested in animal models^18–23^. Other studies have examined non-modified peptide-based adjuvanted vaccines, as well as antibodies specific to non-modified linear and conformational tau epitopes as potential anti-tau immunotherapies^24–29^.

Previously we developed a MultiTEP-based protein vaccine targeting the N-terminus of tau, AV-1980R and showed that this approach induces extremely high titers of anti-tau antibodies^30^. In designing this vaccine, we chose to target amino acids 2–18 of the tau N-terminus as a B cell epitope based on published data showing that this region, comprising phosphatase-activating domain (PAD), is hidden in microtubule bound tau conformations but becomes exposed with tau aggregation^31^. PAD exposure inhibits anterograde fast axonal transport (FAT), and also promotes the polymerization of tau, contributing to toxicity in human tauopathies^32–38^. In this report, we tested for the first time the immunogenicity and therapeutic efficacy of AV-1980R formulated with Advax^CpG^ adjuvant (AV-1980R/A) in the PS19 tau transgenic mouse model and demonstrated that the vaccine generated extremely high titers of anti-tau antibodies that recognized pathological tau in human AD brain tissue and reduced the accumulation of total and certain phospho-tau epitopes in the brains of vaccinated PS19 transgenic mice. Importantly, this approach also led to significant improvements in both cognitive and motor function in immunized mice.

## Results

### Immunogenicity of AV-1980R vaccine in PS19 mice

**Figure 1:**
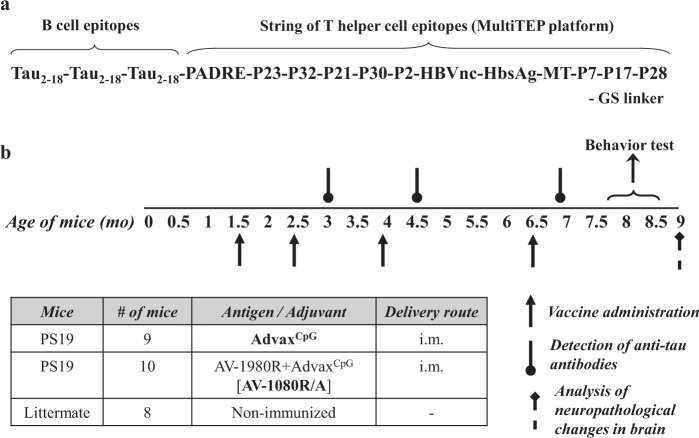
Schematic representations of Protein vaccine construct and experimental design in PS19 transgenic mice. (**a**) Schematic representation of AV-1980 construct encoding 3 copies of tau2-18 fused to MultiTEP, one universal synthetic Th epitope, PADRE and eleven foreign promiscuous Th epitopes from infectious agents. (**b**) Design of experimental protocol in PS19 transgenic mice vaccinated with AV-1980R/A and injected with Advax^CpG^ adjuvant only (control group).

**Figure 2:**
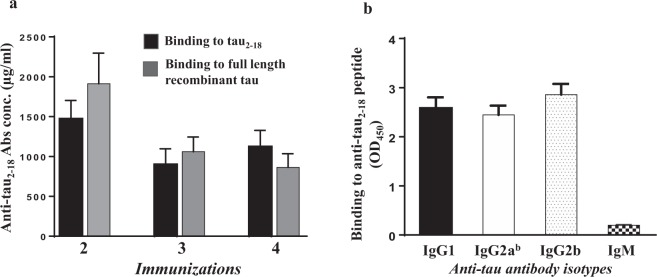
The AV-1980R/A vaccine induced high titers of antibodies in PS19 mice. (**a**) Concentration of anti-tau antibodies in sera was measured by binding to tau_2-18_ peptide and to full length recombinant tau protein in ELISA. (**b**) Isotypes of anti-tau antibodies were analyzed in sera at dilution 1:500 by ELISA. Error bars represent average ± SEM (n = 10 for Advax^CpG^ injected group; n = 9 for AV-1980R/A immunized group).

Tau PS19 mice immunized with AV-1980R (Fig. 1a) formulated with Advax^CpG^ adjuvant (AV-1980R/A, Fig. 1b) produced very high concentrations of anti-tau antibodies after just two immunizations **(**Fig. 2a**)**. After the third and fourth immunizations, mice exhibited slight reductions in antibody titer, although the observed titers remained very high. The resulting antibodies were found to bind equally well to both the tau_2-18_ peptide and full-length recombinant tau protein by ELISA **(**Fig. 2b**)**. An isotype analysis revealed that AV-1980R/A induced a mixed type of immune response, producing equally high levels of IgG1, IgG2^ab^, and IgG2^b^ antibodies, while the level of IgM was negligible **(**Fig. 2c**)**. The ratio of IgG1/IgG2a^b^ in all vaccinated mice was approximately 1, indirectly suggesting an equal contribution of both Th2 and Th1 cytokines to the immune response, which is consistent with the characteristics of Advax^CpG^ ^30^.

**Figure 3:**
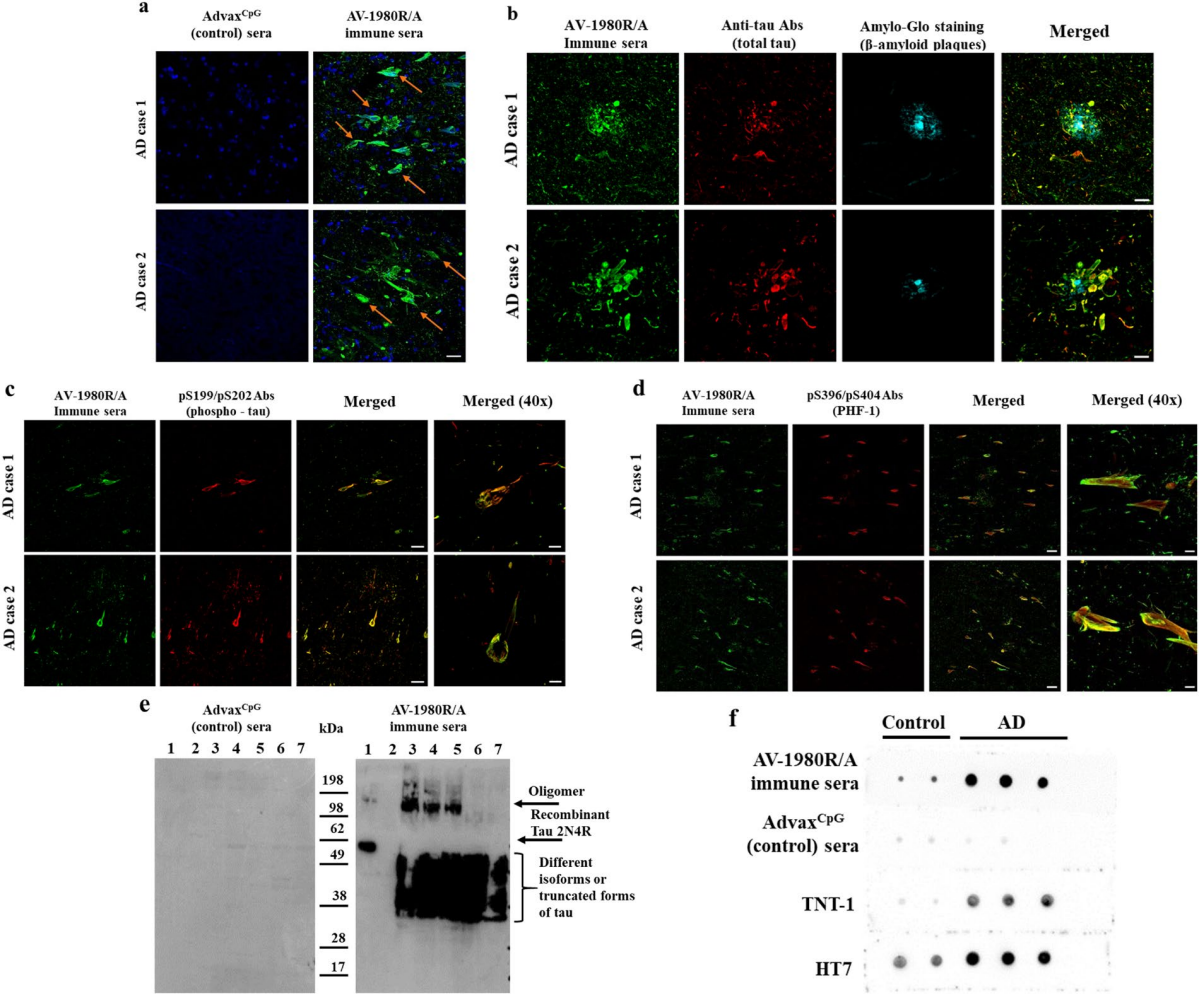
AV-1980R/A vaccine induced functional antibodies recognizing human pathological tau. (**a**) Immune sera, but not sera from adjuvant injected mice bound to the 50 µm brain sections of cortical tissues from two AD cases (scale = 10 µm). (**b–d**) Co-staining of AD brain sections with immune sera and total htau and Amylo-Glo (**b**, scale = 20 µm), pS199/202 (**c**, scale = 40 µm, merge-40x = 10 µm) and pS396/404 (**d**, scale = 50 µm, merge-40x = 10 µm) demonstrated co-localization of tau recognized by anti-tau_2-18_ sera and these antibodies. (**e**) Immune sera, but not sera from adjuvant injected mice also bound to different forms of tau in soluble extracts from four AD cases detected by Western blot. Sera were used at dilution 1:1000. Sera bound full-length recombinant tau, but not tau lacking aa 2-18. Lane 1- Recombinant tau 2N4R; Lane 2 – Recombinant tau 2N4R with deleted aa2-18 (ΔTau2-18); Lane 3-severe AD, tangle stage 6; Lane 4- moderate AD, tangle stage 5; Lane 5-mild AD tangle stage 5, Lane 6 – MCI, tangle stage 2; Lane 7-Control non-AD, stage 2. (**f**) Using non-denaturing Dot Blots, AV-1980R immunized sera as well as commercial TNT-1 mAb specific to N-terminus of Tau (epitope aa 7–12) preferentially bound to soluble tau in AD brain lysates but showed minimal immunoreactivity in controls. Of note, the HT7 Ab which detects total tau, recognized non-denatured tau in both control and AD brains.

Next, we analyzed the functionality of antibodies generated by AV-1980R/A, analyzing their ability to bind pathological tau in AD brains. As shown in Fig. 3a, immune sera from mice vaccinated with AV-1980R/A bound to both neuropil threads (NTs) and neurofibrillary tangles (NFT) in human brain tissue from two AD cases **(**Fig. 3a). Of note, only nonspecific background binding to the brain tissue for AD cases were observed when we used sera from control Tg mice injected with adjuvant only. Co-staining of AD brain sections with immune sera and various anti-tau antibodies (total htau, pS199/202 and pS396/404) further demonstrated co-localization of tau recognized by anti-tau_2-18_ sera and these antibodies within NFTs and dystrophic neurites (Fig. 3b–d). In addition, we utilized Amylo-Glo staining which detects fibrillar β-amyloid and demonstrated the close proximity of AV-1980R/A immunoreactive dystrophic neurites with amyloid plaques (Fig. 3b).

Although both soluble and fibrillar tau may contribute to neuronal and synaptic dysfunction and loss, growing evidence suggests that soluble tau oligomers are the more toxic form and the accumulation of oligomers at the synapse may be critical for neurodegeneration^39–41^. Therefore, binding to different species of tau was shown in soluble fractions of brain homogenates from AD cases at different tau tangle stages. Immune sera, but not control sera bound various monomeric and oligomeric forms of tau in brain extracts from non-AD control (tangle stage 2), MCI (tangle stage 1), mild (tangle stage 4), moderate (tangle stage 5) and severe (tangle stage 6) AD cases detected by western blot (Fig. 3e). In addition, as expected, antibodies bound to full-length recombinant tau, but not to tau that lacks the tau2-18 region. Under non-denaturing conditions (dot blot), AV-1980R/A immune serum showed increased selectively for the soluble fraction of AD brain extracts (Fig. 3f) indicating that PAD is likely more exposed and accessible in AD than control brains when techniques that preserve protein conformation are used.

### Motor and cognitive changes in PS19 mice after immunization with AV-1980R

**Figure 4:**
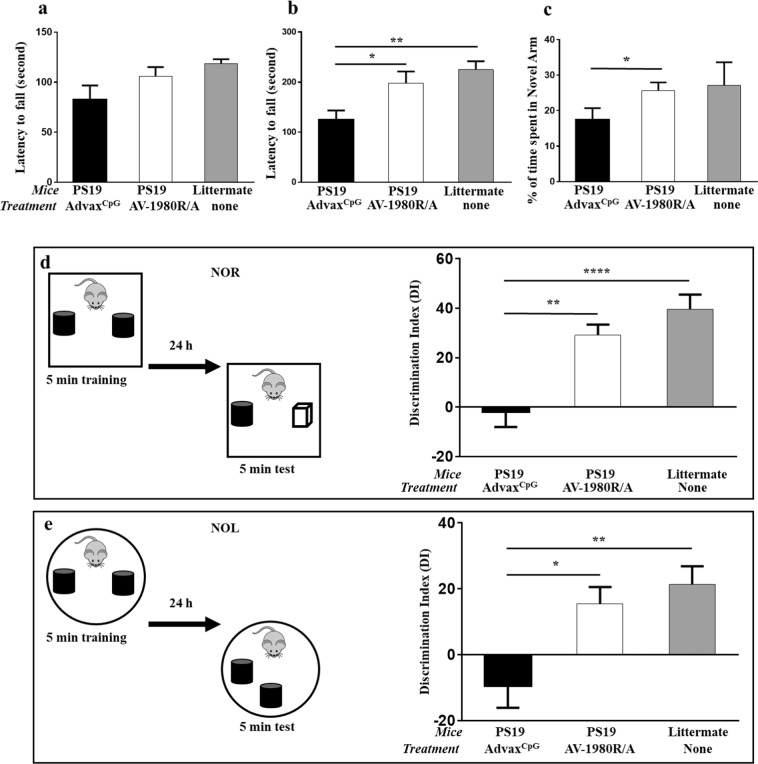
Vaccination with AV-1980R/A improved both motor and cognitive function in PS19 mice. Motor skills of mice were tested in Fixed rotarod (**a**) and accelerated speed rotarod (**b**). Cognitive functions of mice were tested in Y maze alternation test (**c**) and in novel object recognition (**d**) and novel place recognition (**e**) test. Error bars represent average ± SEM (n = 10 for Advax^CpG^ injected group; n = 9 for AV-1980R/A immunized group and n = 8 for non-injected littermate). Statistical significances were calculated using ANOVA test (*P < 0.05, **P < 0.01 and ****P < *0.0001*).

PS19 mice display age-associated motor deficits that progress to paralysis beginning at 10 months of age^42^. The rotarod test is one of the most widely used tests for measuring motor coordination and balance in mouse models. Therefore, PS19 mice were examined for their ability to run on a rotarod, and two tests, fixed and accelerated, were performed at 8 months of age. As shown in Fig. 4a, although, wildtype mice ran longer on a fixed rotarod, then adjuvant injected control PS19 mice, this difference was not significant. However, we did observe a significant impairment of motor function in the acceleration test in PS19 mice that were injected with adjuvant, compared to wildtype control littermates, and importantly, vaccination significantly improved the motor performance of these mice (*p* < *0*.*05*, Fig. 4b).

Alternation tasks measure the disposition of rodents to explore new environments and are used to evaluate the spatial working memory and exploratory behavior^43^. The Y-maze test was used for analyzing alternation. The percentage of time spent in the novel arm was significantly higher for vaccinated mice compared with age-matched control mice injected with adjuvant (28,68% ± 7.22 vs. 17.38% ± 9.93; *p* < *0*.*05*, Fig. 4c).

To study how the immunization with AV-1980R/A affects cognition, PS19 mice were also tested in novel object recognition (NOR) and novel place recognition (NPR) paradigms, which rely on cortical and hippocampal function respectively, both areas strongly affected by AD pathology^44–46^. This analysis demonstrated that vaccination of PS19 mice with AV-1980R/A resulted in a significant increase in the exploration of a non-familiar object as indicated by the increased discrimination index compared with mice injected with adjuvant only (*p* < *0.01*; Fig. 4d). Similar significant improvements were detected during NPR task performance (p < 0.05; Fig. 4e).

### Changes of tau pathology in PS19 mice after immunization with AV-1980R

**Figure 5:**
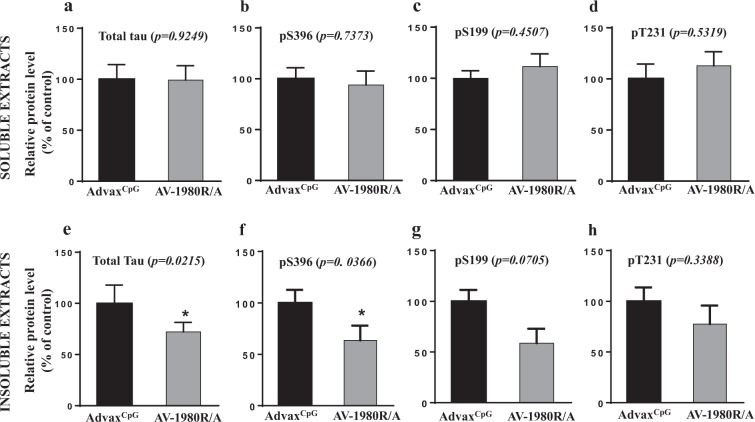
Effect of AV-1980R/A vaccination on tau proteins in PS19 mice. Levels of human total tau protein (**a**,**e**) and several phosphorylated tau species (**b**–**d**,**f**–**h**) in brain soluble (**a–d**) and insoluble (**e–h**) extracts were analyzed by ELISA. Error bars represent average ± SEM (n = 9 for both groups).

PS19 mice begin to develop NFT-like tau pathology within specific brain regions by six months of age. To understand the impact of immunization on tau pathology and the relationship between this pathology and behavioral improvement in vaccinated mice, we next analyzed soluble and insoluble brain homogenates using a sensitive and human tau-specific ELISA. As shown in Fig. 5 we detected significant decreases in insoluble total tau (Fig. 5e) and tau phosphorylated at position pS396 (41% reduction) (Fig. 5f) in brain homogenates of AV-1980R vaccinated mice compared with control animals. We also observed a trend toward reduction of insoluble tau, phosphorylated at positions pS199 (36% reduction) (Fig. 5g) and pT231(22% reduction) (Fig. 5h), but this trend did not reach statistical significance. In contrast, no reduction was detected in analyzed species of tau in soluble fraction of brain homogenates (Fig. 5a–d).

**Figure 6:**
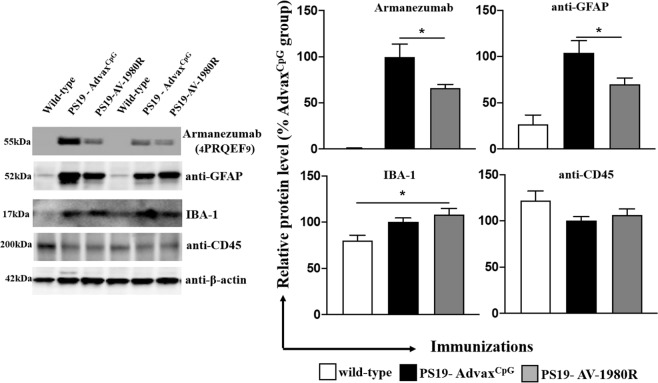
Vaccination with AV-1980R/A significantly decreased PAD-positive Tau and astrogliosis in brains of PS19 mice. The levels of PAD-positive tau protein, GFAP, IBA-1 and CD45 proteins in the soluble fraction of the brain extracts were analyzed by Western blotting and quantitatively determined by densitometric analysis with normalization against β-actin. The relative protein level in the brains of vaccinated mice is presented as a percentage of the protein level in the brains of mice injected with Advax^CpG^. Error bars represent average ± SEM of (n = 9 for both groups).

To assess the effect of AV-1980R/A vaccination on the inflammatory milieu, we next measured the expression of glial fibrillary acidic protein (GFAP), Ionized calcium binding adaptor molecule 1 (IBA-1) and CD45 in brain extracts of vaccinated and control mice. In parallel, using a humanized mAb specific to tau_4-8_, we also demonstrated a significant decrease of PAD-exposed Tau in the brains of vaccinated mice. Interestingly, whereas control vaccinated PS19 mice exhibit a significant increase in GFAP expression versus wild-type controls, indicative of a heightened astrocytic activation state, AV-1980R/A vaccinated PS19 mice exhibited a significant reduction in GFAP. These data suggest that AV-1980R/A mediated reduction in pathological tau likely leads to a corresponding diminution of astrocyte activation. In contrast, no differences were detected in the expression of IBA-1 or CD45 between wild-type and control-vaccinated PS19 mice, although AV-1980R/A vaccinated PS19 mice did exhibit increased IBA-1 levels in comparison to wild-type mice. Taken together, these data suggest that tau pathology primarily promotes an astrocytic response in 9-month old female PS19 mice and that vaccination can in turn reduce this response. However, AV-1980R/A vaccination can also modulate the microglial response to pathology, potentially via Fc-mediated microglial signaling (Fig. 6).

## Discussion

Over the past two decades immunotherapy for the treatment of AD has been actively pursued as a promising approach to promote the clearance of pathological proteins. Since the late nineties, when the “amyloid cascade hypothesis” was initially proposed, immunotherapy studies were primarily directed at the reduction of Aβ pathology. Despite recent clinical failures, this strategy may still offer some promise as a potential preventive approach to block the accumulation of oligomeric forms of Aβ if it is initiated in early prodromal stages of AD. In fact, data are accumulating that support Aβ-targeting therapeutic intervention at very early stages of the disease (Thal amyloid phase 0–1), when patients still do not have significant cortical NFT pathology, or even in healthy people at risk of developing AD^47^. Importantly, many studies have now shown that tau pathology correlates more closely than Aβ pathology with progression of neurodegeneration in AD, and suggested that the accumulation of NFT mediates some of the effects of amyloid-β pathology on cognitive decline^9,47–49^. However, the development of immunotherapy approaches that target tau is challenging due to the enormous diversity of the pool of physiological and pathological tau molecules and the substantial overlap between these pools. Another challenge is the primarily intracellular localization of tau, although recent reports have provided evidence of a small extracellular pool of tau that likely mediates propagation from cell to cell^50–53^. Remarkably, pre-clinical anti-tau immunotherapy studies have demonstrated that various antibodies reduced tau-like pathology in animal models. These data have provided support for several passive and two active vaccination clinical trials in the USA and EU^54–56^. Several mechanisms have been implicated for antibody mediated reduction of pathological tau. Firstly, anti-tau antibodies within the periphery could sequestrate tau proteins from the CNS^57^. Secondly, a small fraction of antibodies may pass the BBB and bind to extracellular aggregates triggering microglia-mediated removal of tau from the CNS^57,58^. Antibody-mediated clearance of extracellular tau may then promote secretion of intracellular tau through a shift in equilibrium, thus indirectly clearing the intracellular tau aggregates^59^. Binding of antibodies to extracellular tau may change the pathological conformation to impair propagation^60^ or protect cells by blocking the internalization of tau^61^. In addition, it is likely that endosomal-lysosomal pathway is also involved in antibody-mediated clearance of intracellular tau aggregates^58^. Regardless of the exact mechanism of action of anti-tau antibodies, pre-clinical studies have shown that active and passive vaccines targeting different phosphorylated or unmodified tau peptides can initiate the removal of tau aggregates from the central nervous system, reduce or eliminate the spread of tau pathology through the brain, and improve cognitive function in various tau/Tg mouse models^54^. However, these studies have showed that antibody-mediated reduction of pathological tau is more complicated than removal of Aβ and that choosing the right epitopes, and generating high concentrations of antibodies, are also more critical^24,54^.

We previously reported that a DNA-based AV-1980D vaccine composed of three copies of the N-terminal peptide (2–18aa) of tau fused to string of 12 foreign Th epitopes (MultiTEP) induced anti-tau antibodies in the THY-Tau22 transgenic mouse model, which in turn significantly reduced both soluble and insoluble total tau and certain species of phosphorylated tau in the brains of vaccinated mice^62^. Of note, we reported earlier that the same vaccine based on recombinant protein was significantly more immunogenic in wildtype mice when formulated in Advax^CpG^ ^30^. Thus, we sought to test this adjuvanted protein counterpart of our DNA vaccine in an aggressive mouse model of tauopathy, PS19 mice. These mice expressing a tau transgene with the P301S mutation, which causes frontotemporal dementia in human patients^63–65^, develop progressive tau pathology and neurodegeneration with early synaptic deficits, as well as neuroinflammation^42^.

In this strain of transgenic mice of H-2^b^ immune haplotype, AV-1980R/A induced extremely high titers of antibodies, that in some animals exceeded the mg/ml level (5086 µg/ml) and were not lower than 276 µg/ml. This was expected, since at least 6 Th epitopes in the MultiTEP platform are recognized by H-2^b^ MHC class II molecules, inducing strong T cell responses that provide help to tau-specific B cells^30^. In addition, AV-1980R was formulated with delta-inulin based adjuvant Advax^CpG^, which, as we showed previously, was superior to any other adjuvant approved for human use or tested in clinical trials^30^.

The generated IgG antibodies had mixed isotypes with an IgG1/IgG2a^b^ ratio of about 1, which indirectly indicates that the vaccine induced a balanced Th1/Th2 type immune response. This was expected given that the delta inulin is a Th2 adjuvant, and by adding low concentration of CpG (0.1%) in the formulation significantly enhances the immune responses while simultaneously shifting it towards the Th1 type. It should be noted that acute pro-inflammatory responses (IFN-γ + SFC) generated by AV-1980R/A are anticipated to be safe, since Th epitopes in this platform are all foreign and are not expressed in the brain. Moreover, it could be beneficial for AD treatment as well, since the effectiveness of TLR4 agonist MPLA has been shown in AD and PD mouse models. MPLA reduced Aβ load in the brain of APP/Tg mice and enhanced cognitive function^66^, as well as increased the uptake of α-syn by microglia in α-Syn/Tg mice, reduced intracellular α-syn aggregation and reduced motor deficits^67^.

Earlier, we developed monoclonal antibodies specific for the tau4-8 epitope and showed that it binds to both oligomeric tau and NFT in AD brain^29^. The AV-1980R/A vaccine-induced polyclonal antibodies may be specific for different epitopes in tau2-18 and, therefore, bind to the pathological tau differently than MoAb. In addition, it was shown that PAD-exposure is an early event in AD progression, which disappears in later stages of tangle evolution^38^. To characterize the polyclonal antibodies from this point we analyzed binding with tau tangles in brain sections from AD cases, as well as binding to soluble tau in brain extracts from AD cases at different tangle stages. We showed that immune sera bound to NFTs and NTs in brain sections, as well as various forms of tau, monomeric and oligomeric, in the soluble fraction of brain homogenates from AD patients similarly as MoAb.

PS19 mice model several aspects of human tauopathy and have been reported to exhibit behavioral impairments including sensorimotor deficits and deficits in spatial learning and memory^68–70^. To ensure that PAD-specific antibodies can reduce these behavioral deficits we tested PS19 mice in various behavioral tasks.

Importantly, we observed significant improvement of both sensorimotor and cognitive functions in vaccinated mice compared with age-matched controls. Cognitive function was tested in Y maze alternation test and NOR/NPR tests. It is believed that many parts of the brain, including the hippocampus, septum, basal forebrain, and prefrontal cortex, are involved in alternation task. NPR primarily evaluates spatial learning, which mainly relies on hippocampal activity, while NOR test evaluates non-spatial learning of object identity, which relies on multiple brain regions^71–73^. Therefore, our data suggest that antibodies generated by AV-1980R/A were able to modulate tau pathology and improve corresponding functional impairments within several brain regions. Behavioral improvement was associated with significant decrease of insoluble tau phosphorylated at position S396. We also observed a trend towards decreased insoluble tau phosphorylated at positions S199 (36% reduction) and T231 (22% reduction). Interestingly, we previously showed that antibodies generated by DNA based vaccination in THY-Tau-22 mice decreased both soluble and insoluble total, and certain species of phosphorylated tau^62^. In PS19 mice we detected the reduction of insoluble tau only. This data coincides with data shown by Yanamandra *et al*. after passive administration of monoclonal antibodies specific to tau25–30 in PS19 mice^24^. Such differences may reflect the characteristics of pathology in mouse models expressing hTau carrying one (P301S in PS19 mice)^42^ or two mutations (G272V and P301S in THY-Tau22 mice)^74^. In any case, these data indicate the ability of anti-tau2-18 antibodies to reduce various types of phosphorylated pathological tau, probably depending on differences in the state and magnitude of pathology. Taken together, these results provide further support for the potential translation of this promising vaccine toward human clinical trials.

In summary, our pre-clinical data reveal a very robust antibody response coupled with therapeutic efficacy and significant reductions in insoluble tau. These findings suggest that the AV-1980R vaccine, which is based on the MultiTEP platform and formulated in a safe and strong adjuvant Advax^CpG^, could potentially be used to induce strong immune responses in a broad population of vaccinated subjects with high MHC class II genes polymorphisms.

## Methods

### Mice

In this study we used female PS19 mice expressing the P301S mutant form of human microtubule-associated protein Tau (MAPT), under the control of the mouse prion protein promoter (Prnp)^42^. These mice display tau seeding activity first detected at 1.5 months of age. They progressively develop neurofibrillary tangles in the hippocampus, neocortex, amygdala, brain stem and spinal cord beginning at about six months of age^42^. All animals were housed in a temperature and light cycle-controlled facility, and their care was under the guidelines of the National Institutes of Health and an approved Institutional Animal Care and Use Committee (IACUC) protocol at University of California, Irvine.

### Epitope vaccine and purification of protein

To prepare a recombinant protein, minigene encoding 3Tau_2-18_-MultiTEP was cloned into the *E. coli* expression vector pET24a (for AV-1980R; Novagen, MA) in frame with 6xHis-Tag at the C-terminus (Fig. 1a). DNA sequencing was performed to confirm that the generated plasmid contained the correct sequences. Recombinant protein was purified from *E. coli* BL21 (DE3) cells transformed with pET24a/3Tau_2-18_-MultiTEP plasmid as described^30,75^. The final recombinant protein was analysed in 10% Bis-Tris gel electrophoresis (NuPAGE Novex Gel, Invitrogen, CA). Proteins were visualized by Coomassie dye and specificity of bands confirmed by Western Blot (WB) using anti-Tau_2-18_ 1C9 monoclonal antibody^30^. Endotoxin levels were measured using E-TOXATE kits, as recommended by the manufacturer (Sigma, St Louis, MO).

### Immunizations

Female, 1.5-month-old PS19 mice were immunized with AV-1980R (Fig. 1) (20 µg/per mouse/per injection; n = 10) formulated with Advax^CpG^ adjuvant (1 mg/mouse/injection) as previously described^30^. The control group of PS19 mice were injected with Advax^CpG^ adjuvant only (n = 9). All mice were injected four times, intramuscularly. Littermate mice (n = 8) were used during the behavior testing. Sera were collected 14 days after second, third and fourth immunizations, and anti-tau antibody responses were analyzed. At age of 8-month behavior tests were performed. Mice were terminated at age of 9-month-old and brains were collected for biochemical analysis.

### Detection of tau-specific antibodies and isotyping

The concentrations of anti-tau antibodies in serum were quantified by ELISA, as previously described^30^. Briefly, to measure anti-tau antibody concentration plates were coated with 1 µg/per well tau2-18 peptide (GenScript, NJ) or full-length recombinant tau and it was calculated using a calibration curve generated with polyclonal anti-tau2-18 antibodies purified from AV-1980R/A vaccinated mouse sera (The Institute for Molecular Medicine, Huntington Beach, CA). HRP-conjugated anti-mouse IgG (Jackson ImmunoResearch Laboratories, ME) was used as a secondary antibody. HRP-conjugated anti-IgG1, IgG2ab, IgG2b and IgM specific antibodies (Bethyl Laboratories, Inc., TX) were used to characterize the isotype profiles of anti-tau antibodies in individual sera at 1:500 dilutions.

### Detection of tau tangles and β-amyloid plaques in human brain tissues by IHC and confocal microscopy

Sera from mice immunized with AV-1980R/A, as well as injected with Advax^CpG^ were screened for the ability to bind to human tau tangles using 40 µm brain sections of formalin-fixed cortical tissues from severe AD cases (generously provided by the UC Irvine Alzheimer’s Disease Research Center (ADRC) Tissue Repository) using immunohistochemistry as described previously^62^. In addition, AD brain sections were stained with several commercial antibodies: anti-human tau (Agilent, CA), anti-phospho tau [pS199; pS202; pS396; pS404] (all from Abcam, UK) and Amylo-Glo (Biosensis, Australia). Sections were imaged using an Olympus FX1200 confocal microscope.

### Preparation of brain homogenates from AD cases and controls, Western blot and Dot blot analysis

Preparation of brain homogenates, Western blot (WB) and Dot blot (DB) analysis were performed as previously described^30,62^. Briefly, 0.2 g of brain tissue from four different AD cases were homogenized in 0.4 ml TBS buffer with Halt™ Protease and Phosphatase Inhibitor Cocktail (100X, Thermo Scientific, CA), then centrifuged at 6400xg for 15 minutes at +4 °C. Supernatants (soluble fractions) were collected and stored at −80 °C for further analysis. For WB soluble fractions applied to electrophoresis on NuPAGE 4–12% Bis-Tris gel in MES buffer under reducing conditions (Invitrogen, CA) and electrotransferred onto nitrocellulose membrane (GE Healthcare, NJ). Tau were visualized by incubating with sera (dilution at 1:1000) from mice immunized with AV-1980R/A and injected with Advax^CpG^ only followed by HRP-conjugated anti-mouse IgG (Santa Cruz Biotechnology, CA). For DB assay the same extracts were applied to membrane (1 μg). Proteins were detected using sera from mice immunized with AV-1980R/A and control mice injected with Advax^CpG^ only, TNT-1 (Millipore, MA), HT7 (Life Technology, CA) antibodies. All primary antibodies were used at concentration of 1 μg/ml, serum was used at dilution 1:2500. Bovine anti-mouse HRP-conjugated secondary antibody was used (Santa Cruz Biotechnology, CA).

### Behavior tests

All behavioral experiments were run by an investigator who was blinded to genotype and treatment. Results were then de-coded during statistical analysis by a second independent investigator.

#### Rotarod test

The rotarod is an automated apparatus with a 3–7 cm diameter “grooved rod”, speed controls, and a lever that triggers the timer to stop, once the timer is stopped the mouse falls from the rod. For *fixation rotarod test* the mice were trained for three days in raw at 12 rpm to stay on the fixed speed rotarod for 2 min. If animals fall off, they were placed back onto the spinning rod, so as to learn the task. Twenty-four hours later after the last training session, mice were tested for 4 consecutive trials at 12 rpm for 2 min and latency to fall off of the rotating rod is measured and data was presented as average of four trials. For *acceleration test* rotarod speed increased in rpm (from 4 to 40) over the entire testing trial (5 minutes).

#### Y-maze

The experimental apparatus consisted of a Y-maze made of clear Plexiglas as described previously^76^ with some modifications. The apparatus is a three-arm horizontal maze in which the arms are arranged at 120° angles to each other. Two arms (B and C) are 15 cm in length and one arm (A) is 20 cm long. All arms are 5 cm in width, and the walls are 12 cm high. The maze walls and floor are made opaque with 0.5 cm thick poster board. The poster board is attached to the outside walls of the maze and a separate piece is placed under the maze base making the floor opaque as well. Mice were habituated to the room for 1 h prior to the test, in the dark phase. The maze was placed in a dimly lit room. Mice are initially placed in the long arm (A) with their head facing the maze arms. The animals were then given 7 minutes to explore the maze, while an overhead video camera tracks their behavior. An alternation is defined as consecutive entries into all three arms (i.e., ABC, CAB, or BCA, but not BAB).

#### Novel object and novel place recognition tests

Novel object and novel place recognition tests were used to evaluate cognition as previously described^46,77^. All combinations of locations and objects were balanced across trials to eliminate bias. Training and testing trials were videotaped and analyzed by individuals blind to the treatment condition. A mouse was scored as exploring an object when its head was oriented toward the object and within a distance of 1 cm, or when its nose was touching the object. The relative exploration time was recorded and expressed by a discrimination index (DI = (t_novel_ − t_familiar_)/(t_novel_ + t_familiar_) × 100%).

### Biochemical analyses

Right hemispheres of mouse brain, previously frozen on dry ice and stored at −80 °C, were crushed on dry ice using mortar and pestle, then homogenized in solution of T-PER (Thermo Scientific, MA) and phosphatase and protease inhibitor mixtures (Thermo Scientific, MA and Roche, CA) and processed as previously described^62,78,79^. Concentrations of human total and phosphorylated Tau in samples (soluble and insoluble brain extracts) were determined by Tau (total) Human ELISA kit, Tau [pS396] Human ELISA Kit, Tau [pS199] Human ELISA Kit, and Tau [pT231] Human ELISA Kit (all from ThermoFisher Scientific, MA), according to the manufacturer’s instructions.

Soluble SDS-PAGE WB was performed following standard protocols as previously described^62,78,79^. Primary antibodies used for WB analysis included the following: Armanezumab (1:2000; Institute for Molecular Medicine, Huntington Beach, CA), anti-GFAP (1:400; Millipore-Sigma, MO), IBA-1 (1:200; FUJIFILM Wako Chemicals U.S.A. Corp, VA) and anti-CD45 (1:400; Bio-Rad, CA). All blot membranes were also labeled with anti-β-actin antibodies (1:1000; Millipore-Sigma, MO) as loading control.

### Statistical analysis

Statistical parameters (mean, standard deviation (SD), standard errors (SE), significant difference, etc.) were calculated using the Prism 6 software (GraphPad Software, Inc., La Jolla, CA). Statistically significant differences were examined using a two-tailed t-test or ordinary one-way ANOVA Tukey’s multiple comparisons test (a P value of less than 0.05 was considered significant).

## References

[1] Berk C, Paul G, Sabbagh M (2014). Investigational drugs in Alzheimer’s disease: current progress. Expert Opin Investig Drugs.

[2] Wang J, Gu BJ, Masters CL, Wang YJ (2017). A systemic view of Alzheimer disease - insights from amyloid-beta metabolism beyond the brain. Nat Rev Neurol.

[3] Golde TE, Schneider LS, Koo EH (2011). Anti-abeta therapeutics in Alzheimer’s disease: the need for a paradigm shift. Neuron.

[4] Folch J (2016). Current Research Therapeutic Strategies for Alzheimer’s Disease Treatment. Neural Plast.

[5] Selkoe DJ (2019). Alzheimer disease and aducanumab: adjusting our approach. Nat Rev Neurol.

[6] Hardy J, Allsop D (1991). Amyloid deposition as the central event in the aetiology of Alzheimer’s disease. Trends Pharmacol Sci.

[7] Schneider LS (2014). Clinical trials and late-stage drug development for Alzheimer’s disease: an appraisal from 1984 to 2014. J Intern Med.

[8] Selkoe DJ (2018). Light at the End of the Amyloid TunnelPublished as part of the Biochemistry series “Biochemistry to Bedside”. Biochemistry.

[9] Nelson PT (2012). Correlation of Alzheimer disease neuropathologic changes with cognitive status: a review of the literature. J Neuropathol Exp Neurol.

[10] Wischik CM, Harrington CR, Storey JM (2014). Tau-aggregation inhibitor therapy for Alzheimer’s disease. Biochem Pharmacol.

[11] Savage MJ (2014). A sensitive abeta oligomer assay discriminates Alzheimer’s and aged control cerebrospinal fluid. J Neurosci.

[12] McLean CA (1999). Soluble pool of Abeta amyloid as a determinant of severity of neurodegeneration in Alzheimer’s disease. Ann Neurol.

[13] Lue LF (1999). Soluble amyloid beta peptide concentration as a predictor of synaptic change in Alzheimer’s disease. Am J Pathol.

[14] Tomic JL, Pensalfini A, Head E, Glabe CG (2009). Soluble fibrillar oligomer levels are elevated in Alzheimer’s disease brain and correlate with cognitive dysfunction. Neurobiol Dis.

[15] Zilka N (2012). Who fans the flames of Alzheimer’s disease brains? Misfolded tau on the crossroad of neurodegenerative and inflammatory pathways. J Neuroinflammation.

[16] Pedersen JT, Sigurdsson EM (2015). Tau immunotherapy for Alzheimer’s disease. Trends Mol Med.

[17] Medina Miguel (2018). An Overview on the Clinical Development of Tau-Based Therapeutics. International Journal of Molecular Sciences.

[18] Asuni AA, Boutajangout A, Quartermain D, Sigurdsson EM (2007). Immunotherapy targeting pathological tau conformers in a tangle mouse model reduces brain pathology with associated functional improvements. J Neurosci.

[19] Boutajangout A, Quartermain D, Sigurdsson EM (2010). Immunotherapy targeting pathological tau prevents cognitive decline in a new tangle mouse model. J Neurosci.

[20] Bi M, Ittner A, Ke YD, Gotz J, Ittner LM (2011). Tau-targeted immunization impedes progression of neurofibrillary histopathology in aged P301L tau transgenic mice. PLoS One.

[21] Troquier L (2012). Targeting phospho-Ser422 by active Tau Immunotherapy in the THYTau22 mouse model: a suitable therapeutic approach. Curr Alzheimer Res.

[22] Goni F (2013). Immunomodulation targeting of both Abeta and tau pathological conformers ameliorates Alzheimer’s disease pathology in TgSwDI and 3xTg mouse models. J Neuroinflammation.

[23] Theunis C (2013). Efficacy and safety of a liposome-based vaccine against protein Tau, assessed in tau.P301L mice that model tauopathy. PLoS One.

[24] Yanamandra K (2015). Anti-tau antibody reduces insoluble tau and decreases brain atrophy. Ann Clin Transl Neurol.

[25] Novak, P., Zilka, N., Kontsekova, E., Ondrus, M. & Novak, M. In *Alzheimer’s Association International Conference* (2016).

[26] Rasool S, Martinez-Coria H, Wu JW, LaFerla F, Glabe CG (2013). Systemic vaccination with anti-oligomeric monoclonal antibodies improves cognitive function by reducing Abeta deposition and tau pathology in 3xTg-AD mice. J Neurochem.

[27] Dai CL, Tung YC, Liu F, Gong CX, Iqbal K (2017). Tau passive immunization inhibits not only tau but also Abeta pathology. Alzheimers Res Ther.

[28] Castillo-Carranza DL (2014). Specific targeting of tau oligomers in Htau mice prevents cognitive impairment and tau toxicity following injection with brain-derived tau oligomeric seeds. J Alzheimers Dis.

[29] Agadjanyan MG (2017). Humanized monoclonal antibody armanezumab specific to N-terminus of pathological tau: characterization and therapeutic potency. Mol Neurodegener.

[30] Davtyan H (2016). Alzheimer’s disease Advax(CpG)- adjuvanted MultiTEP-based dual and single vaccines induce high-titer antibodies against various forms of tau and Abeta pathological molecules. Sci Rep.

[31] Jeganathan S, von Bergen M, Brutlach H, Steinhoff HJ, Mandelkow E (2006). Global hairpin folding of tau in solution. Biochemistry.

[32] Ward SM, Himmelstein DS, Lancia JK, Binder LI (2012). Tau oligomers and tau toxicity in neurodegenerative disease. Biochem Soc Trans.

[33] Morfini GA (2009). Axonal transport defects in neurodegenerative diseases. J Neurosci.

[34] Horowitz PM (2004). Early N-terminal changes and caspase-6 cleavage of tau in Alzheimer’s disease. J Neurosci.

[35] Gamblin TC, Berry RW, Binder LI (2003). Modeling tau polymerization *in vitro*: a review and synthesis. Biochemistry.

[36] Kanaan NM (2012). Phosphorylation in the amino terminus of tau prevents inhibition of anterograde axonal transport. Neurobiol Aging.

[37] Kanaan NM (2011). Pathogenic forms of tau inhibit kinesin-dependent axonal transport through a mechanism involving activation of axonal phosphotransferases. J Neurosci.

[38] Combs B, Hamel C, Kanaan NM (2016). Pathological conformations involving the amino terminus of tau occur early in Alzheimer’s disease and are differentially detected by monoclonal antibodies. Neurobiol Dis.

[39] Kopeikina KJ, Hyman BT, Spires-Jones TL (2012). Soluble forms of tau are toxic in Alzheimer’s disease. Transl Neurosci.

[40] Guerrero-Munoz MJ, Gerson J, Castillo-Carranza DL (2015). Tau Oligomers: The Toxic Player at Synapses in Alzheimer’s Disease. Front Cell Neurosci.

[41] Shafiei SS, Guerrero-Munoz MJ, Castillo-Carranza DL (2017). Tau Oligomers: Cytotoxicity, Propagation, and Mitochondrial Damage. Front Aging Neurosci.

[42] Yoshiyama Y (2007). Synapse loss and microglial activation precede tangles in a P301S tauopathy mouse model. Neuron.

[43] Deacon RM, Rawlins JN (2006). T-maze alternation in the rodent. Nat Protoc.

[44] Cohen SJ, Stackman RW (2015). Assessing rodent hippocampal involvement in the novel object recognition task. A review. Behav Brain Res.

[45] Guzman-Ramos K (2012). Restoration of dopamine release deficits during object recognition memory acquisition attenuates cognitive impairment in a triple transgenic mice model of Alzheimer’s disease. Learn Mem.

[46] Petrushina I (2017). Comparison of Efficacy of Preventive and Therapeutic Vaccines Targeting the N Terminus of beta-Amyloid in an Animal Model of Alzheimer’s Disease. Mol Ther.

[47] Murray ME (2015). Clinicopathologic and 11C-Pittsburgh compound B implications of Thal amyloid phase across the Alzheimer’s disease spectrum. Brain.

[48] Bennett DA, Schneider JA, Wilson RS, Bienias JL, Arnold SE (2004). Neurofibrillary tangles mediate the association of amyloid load with clinical Alzheimer disease and level of cognitive function. Arch Neurol.

[49] Giannakopoulos P (2003). Tangle and neuron numbers, but not amyloid load, predict cognitive status in Alzheimer’s disease. Neurology.

[50] Gerson JE, Kayed R (2013). Formation and propagation of tau oligomeric seeds. Front Neurol.

[51] Clavaguera F (2013). “Prion-like” templated misfolding in tauopathies. Brain Pathol.

[52] Yanamandra K (2013). Anti-tau antibodies that block tau aggregate seeding *in vitro* markedly decrease pathology and improve cognition *in vivo*. Neuron.

[53] Frost B, Diamond MI (2010). Prion-like mechanisms in neurodegenerative diseases. Nat Rev Neurosci.

[54] Novak P, Kontsekova E, Zilka N, Novak M (2018). Ten Years of Tau-Targeted Immunotherapy: The Path Walked and the Roads Ahead. Front Neurosci.

[55] Novak P (2017). Safety and immunogenicity of the tau vaccine AADvac1 in patients with Alzheimer’s disease: a randomised, double-blind, placebo-controlled, phase 1 trial. Lancet Neurol.

[56] West T (2017). Preclinical and Clinical Development of ABBV-8E12, a Humanized Anti-Tau Antibody, for Treatment of Alzheimer’s Disease and Other Tauopathies. J Prev Alzheimers Dis.

[57] Gu, J., Congdon, E. E. & Sigurdsson, E. M. Two novel tau antibodies targeting the 396/404 region are primarily taken up by neurons and reduce tau pathology. *J Biol Chem* (2013). 10.1074/jbc.M113.494922 PMC3829157 24089520

[58] Krishnamurthy PK, Deng Y, Sigurdsson EM (2011). Mechanistic Studies of Antibody-Mediated Clearance of Tau Aggregates Using an *ex vivo* Brain Slice Model. Front Psychiatry.

[59] Sigurdsson EM (2009). Tau-focused immunotherapy for Alzheimer’s disease and related tauopathies. Curr Alzheimer Res.

[60] Sarkar M, Kuret J, Lee G (2008). Two motifs within the tau microtubule-binding domain mediate its association with the hsc70 molecular chaperone. J Neurosci Res.

[61] Evans LD (2018). Extracellular Monomeric and Aggregated Tau Efficiently Enter Human Neurons through Overlapping but Distinct Pathways. Cell Rep.

[62] Davtyan H (2017). MultiTEP platform-based DNA epitope vaccine targeting N-terminus of tau induces strong immune responses and reduces tau pathology in THY-Tau22 mice. Vaccine.

[63] Baba Y (2007). Clinical and genetic features of families with frontotemporal dementia and parkinsonism linked to chromosome 17 with a P301S tau mutation. J Neural Transm (Vienna).

[64] Lossos A (2003). Frontotemporal dementia and parkinsonism with the P301S tau gene mutation in a Jewish family. J Neurol.

[65] Morris HR (2001). The genetic and pathological classification of familial frontotemporal dementia. Arch Neurol.

[66] Michaud JP (2013). Toll-like receptor 4 stimulation with the detoxified ligand monophosphoryl lipid A improves Alzheimer’s disease-related pathology. Proc Natl Acad Sci USA.

[67] Venezia S (2017). Toll-like receptor 4 stimulation with monophosphoryl lipid A ameliorates motor deficits and nigral neurodegeneration triggered by extraneuronal alpha-synucleinopathy. Mol Neurodegener.

[68] Takeuchi H (2011). P301S mutant human tau transgenic mice manifest early symptoms of human tauopathies with dementia and altered sensorimotor gating. PLoS One.

[69] Min, S. W. *et al*. Critical role of acetylation in tau-mediated neurodegeneration and cognitive deficits. *Nat Med* (2015). 10.1038/nm.3951 PMC4598295 26390242

[70] Lasagna-Reeves CA (2016). Reduction of Nuak1 Decreases Tau and Reverses Phenotypes in a Tauopathy Mouse Model. Neuron.

[71] Denninger, J. K., Smith, B. M. & Kirby, E. D. Novel Object Recognition and Object Location Behavioral Testing in Mice on a Budget. *J Vis Exp* (2018). 10.3791/58593 PMC6800058 30531711

[72] Balderas I (2008). The consolidation of object and context recognition memory involve different regions of the temporal lobe. Learn Mem.

[73] Dere E, Huston JP, De Souza Silva MA (2007). The pharmacology, neuroanatomy and neurogenetics of one-trial object recognition in rodents. Neurosci Biobehav Rev.

[74] Schindowski K (2006). Alzheimer’s disease-like tau neuropathology leads to memory deficits and loss of functional synapses in a novel mutated tau transgenic mouse without any motor deficits. Am J Pathol.

[75] Davtyan H (2010). DNA prime-protein boost increased the titer, avidity and persistence of anti-Abeta antibodies in wild-type mice. Gene Ther.

[76] Senechal Y, Kelly PH, Dev KK (2008). Amyloid precursor protein knockout mice show age-dependent deficits in passive avoidance learning. Behav Brain Res.

[77] Passos GF (2013). The bradykinin B1 receptor regulates Abeta deposition and neuroinflammation in Tg-SwDI mice. Am J Pathol.

[78] Blurton-Jones M (2009). Neural stem cells improve cognition via BDNF in a transgenic model of Alzheimer disease. Proc Natl Acad Sci USA.

[79] Marsh SE (2016). The adaptive immune system restrains Alzheimer’s disease pathogenesis by modulating microglial function. Proc Natl Acad Sci USA.

